# TRPV1 activation alleviates cognitive and synaptic plasticity impairments through inhibiting AMPAR endocytosis in APP23/PS45 mouse model of Alzheimer’s disease

**DOI:** 10.1111/acel.13113

**Published:** 2020-02-14

**Authors:** Yehong Du, Min Fu, Zhilin Huang, Xin Tian, Junjie Li, Yayan Pang, Weihong Song, Yu Tian Wang, Zhifang Dong

**Affiliations:** ^1^ Pediatric Research Institute Ministry of Education Key Laboratory of Child Development and Disorders National Clinical Research Center for Child Health and Disorders China International Science and Technology Cooperation Base of Child Development and Critical Disorders Chongqing Key Laboratory of Translational Medical Research in Cognitive Development and Learning and Memory Disorders Children’s Hospital of Chongqing Medical University Chongqing China; ^2^ Department of Neurology Chongqing Key Laboratory of Neurology First Affiliated Hospital of Chongqing Medical University Chongqing China; ^3^ Department of Psychiatry Townsend Family Laboratories University of British Columbia Vancouver BC Canada; ^4^ Brain Research Centre University of British Columbia Vancouver BC Canada

**Keywords:** Alzheimer's disease, AMPA receptor endocytosis, capsaicin, learning and memory, long‐term potentiation, TRPV1

## Abstract

Alzheimer's disease (AD) is one of the most common causes of neurodegenerative diseases in the elderly. The accumulation of amyloid‐β (Aβ) peptides is one of the pathological hallmarks of AD and leads to the impairments of synaptic plasticity and cognitive function. The transient receptor potential vanilloid 1 (TRPV1), a nonselective cation channel, is involved in synaptic plasticity and memory. However, the role of TRPV1 in AD pathogenesis remains largely elusive. Here, we reported that the expression of TRPV1 was decreased in the brain of APP23/PS45 double transgenic AD model mice. Genetic upregulation of TRPV1 by adeno‐associated virus (AAV) inhibited the APP processing and Aβ deposition in AD model mice. Meanwhile, upregulation of TRPV1 ameliorated the deficits of hippocampal CA1 long‐term potentiation (LTP) and spatial learning and memory through inhibiting GluA2‐containing α‐amino‐3‐hydroxy‐5‐methyl‐4‐isoxazolepropionic acid receptor (AMPAR) endocytosis. Furthermore, pharmacological activation of TRPV1 by capsaicin (1 mg/kg, i.p.), an agonist of TRPV1, dramatically reversed the impairments of hippocampal CA1 LTP and spatial learning and memory in AD model mice. Taken together, these results indicate that TRPV1 activation effectively ameliorates cognitive and synaptic functions through inhibiting AMPAR endocytosis in AD model mice and could be a novel molecule for AD treatment.

## Graphical abstract

Aging reduces TRPV1 activity to lead to AMPAR endocytosis and active APP processing to promote Aβ deposition. Increased AMPAR endocytosis and Aβ result in LTP impairment and subsequently induce memory deficits. Genetic upregulation or pharmacological activation of TRPV1 is able to reverse AD‐related neuropathologies in AD model mice.

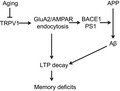

## 1 INTRODUCTION

Alzheimer's disease (AD) is a progressive neurodegenerative disease leading to dementia, which is characterized by extracellular neuritic plaques containing amyloid‐β (Aβ) peptide deposition and intracellular neurofibrillary tangles consisting of hyperphosphorylated tau protein (Davis & Chisholm, 1999; Goedert et al., 1996). Aβ is derived from sequential proteolytic cleavages of Aβ precursor protein (APP) by β‐secretase (BACE1) and γ‐secretase (mainly PS1) (Querfurth & LaFerla, 2010). Synaptic plasticity, a cellular basis for learning and memory (Bliss & Collingridge, 1993; Collingridge, Isaac, & Wang, 2004; Malenka & Nicoll, 1999), can be regulated by changing the number, types, or properties of neurotransmitter receptors in postsynaptic densities (Collingridge et al., 2004). Previous studies have well documented that application of Aβ to cultures and slices disrupts spine morphology (Roselli et al., 2005), while in vivo administration of Aβ impairs synaptic plasticity (Shankar et al., 2008) and cognitive function by binding with AMPARs (Hsieh et al., 2006) or N‐methyl‐D‐aspartic acid receptors (NMDARs) (Kamenetz et al., 2003; Snyder et al., 2005) to cause their internalization. These research progresses support the view that Aβ may be the culprit in the synaptic changes during AD development.

The transient receptor potential vanilloid 1 (TRPV1) channel, a ligand‐gated nonselective cation channel, can be activated not only by exogenous agonist capsaicin, but also by endogenous compounds such as arachidonic acid metabolites and endocannabinoids (Takahashi & Mori, 2011). TRPV1 is initially thought to be a prominent nociceptive ion channel mainly expressed in afferent sensory neurons (Sawamura, Shirakawa, Nakagawa, Mori, & Kaneko, 2017). However, more and more studies have revealed that TRPV1 is also widely expressed in the brain (Mezey et al., 2000; Toth et al., 2005) and involved in several functions such as the modulation of synaptic transmission and plasticity, as well as cognitive functions (Fu, Xie, & Zuo, 2009). For example, TRPV1 activation by capsaicin enhances NMDAR‐dependent CA1 LTP and ameliorates stress‐induced memory decline, which can be blocked by TRPV1 antagonists, capsazepine, and SB366791 (Li et al., 2008). Further genetic studies have shown that TRPV1 knockout significantly reduced the LTP in the hippocampus (Hurtado‐Zavala et al., 2017; Marsch et al., 2007). Collectively, pharmacological activation of TRPV1 enhances LTP, while the genetic elimination of TRPV1 decreases LTP, suggesting a potential target for TRPV1 in promoting hippocampal LTP, and thus protecting learning and memory (Peters, McDougall, Fawley, Smith, & Andresen, 2010).

Synaptic plasticity and cognitive function are significantly impaired in AD, and we therefore hypothesize that TRPV1 activation may alleviate the impairments of LTP and memory during AD progression. Indeed, our recent study has shown that TRPV1 agonist capsaicin can reverse hippocampal CA1 LTP and memory impairments in the Aβ‐induced mouse model of AD (Chen et al., 2017). However, exogenous Aβ treatment is hard to mimic AD development, and so far, little is known about the cellular and molecular mechanism underlying the amelioration of AD symptoms with TRPV1. In the present study, we introduced APP23/PS45 double transgenic model mice of AD and investigated the influence of TRPV1 on the pathological changes of AD and cognitive functions by using a combination of biochemical, electrophysiological, and behavioral assessments.

## 2 RESULTS

### 2.1 TRPV1 reduces Aβ generation in APP23/PS45 mice

**Figure 1:**
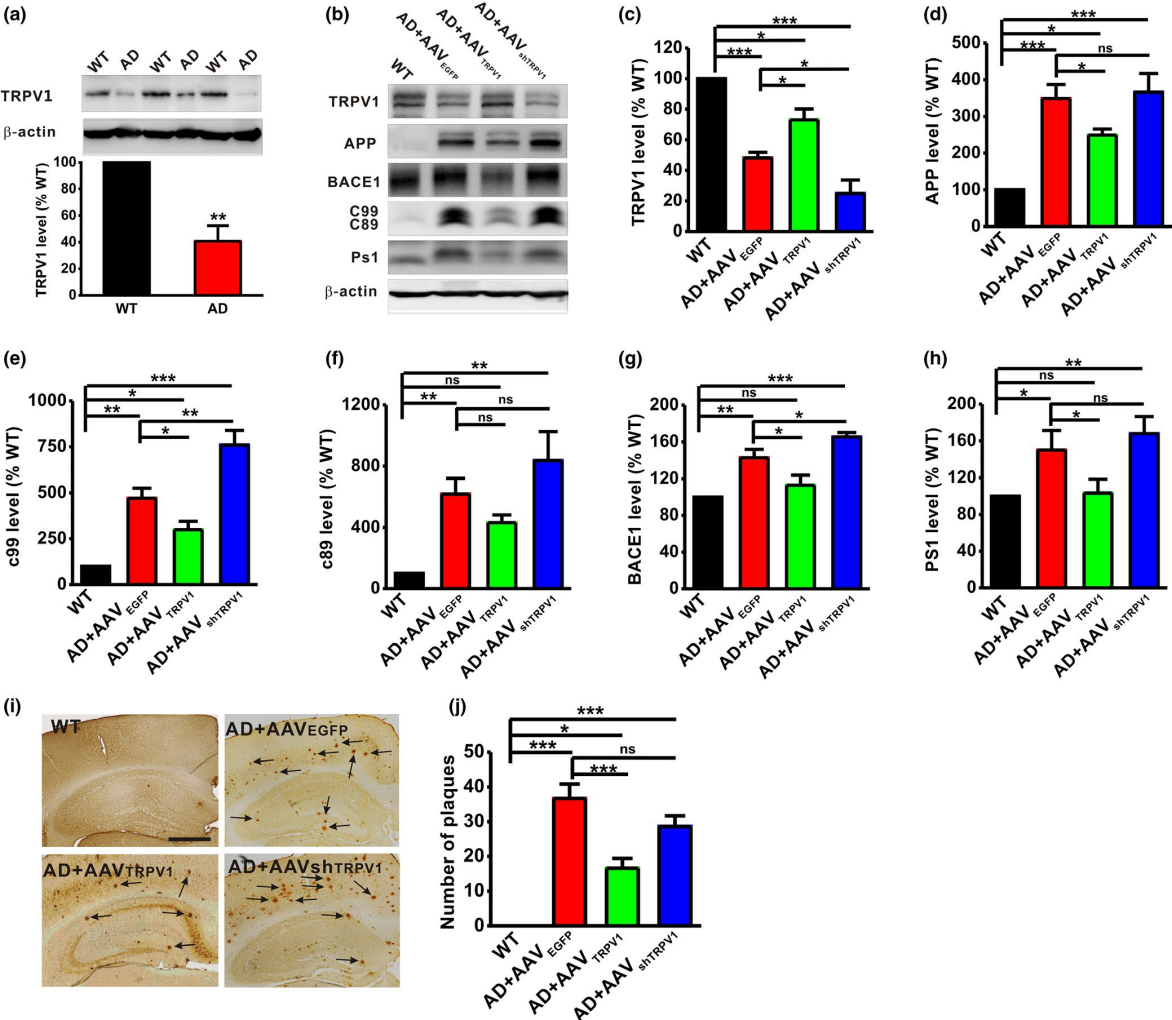
TRPV1 decreases APP processing in APP23/PS45 mice. (a) The protein level of TRPV1 in the brains of WT and APP23/PS45 mice at the age of 4 months. *t* = 40.605, *p* < .001 by unpaired Student's *t* test. *n* = 6 in each group. (b–h) The relative protein levels of TRPV1 (b and c), APP (b and d), c99 (b and e), c89 (b and f), BACE1 (b and g), and PS1 (b and h) are normalized by WT (*n* = 4–9 in each group). One‐way ANOVA: *F*_(3,17)_ = 28.993, *p* < .001 for TRPV1; *F*_(3,29)_ = 13.818, *p* < .001 for APP; *F*_(3,13)_ = 27.499, *p* < .001 for c99; *F*_(3,13)_ =7.871, *p* = .004 for c89; *F*_(3,17)_ =14.975, *p* < .001 for BACE1; and *F*_(3,25)_ = 4.488, *p* = .012 for PS1. (i and j) The number of neuritic plaques detected by immunohistochemistry in the hippocampus of APP23/PS45 mice (*n* = 14–42 slices from 3–9 mice in each group). One‐way ANOVA: *F*_(3,122)_ = 15.092, *p* < .001. Data are expressed as mean ± *SEM*, *p < .05, **p < .01, ***p < .001

To examine the effect of TRPV1 on AD development, we first wanted to determine whether there is an alteration of TRPV1 expression in AD. The results showed that the level of TRPV1 protein was significantly reduced in the brain of APP23/PS45 double transgenic AD model mice at the age of 4 months (AD: 40.65 ± 11.66% relative to WT, *p* < .001 vs. WT; Figure 1a), compared with the age‐matched wild‐type (WT) littermates. Next, we constructed adeno‐associated virus carrying TRPV1 cDNA (AAV_TRPV1_) or TRPV1 shRNA (AAV_shTRPV1_) to investigate the role of TRPV1 in the AD pathogenesis. The virus was microinjected into the hippocampus of APP23/PS45 mice to overexpress or knockdown TRPV1. The results showed that the expression of APP (AD + AAV_EGFP_: 348.68 ± 38.2% relative to WT, *p* < .001 vs. WT; Figure 1b and d) and its C‐terminal fragments (CTFs), including c99 (AD + AAV_EGFP_: 515.35 ± 106.14% relative to WT, *p* = .005 vs. WT; Figure 1b and e) and c89 (AD + AAV_EGFP_: 553.90 ± 84.31% relative to WT, *p* = .009 vs. WT; Figure 1b and f), were significantly increased in the brain of APP23/PS45 mice compared to WT mice. BACE1 (AD + AAV_EGFP_: 142.49 ± 9.37% relative to WT, *p* = .001 vs. WT; Figure 1b and g) and PS1 (AD + AAV_EGFP_: 150.07 ± 21.28% relative to WT, *p* = .037 vs. WT; Figure 1b and h) were also significantly increased in APP23/PS45 mice, while upregulation of TRPV1 (AD + AAV_TRPV1_: 72.98 ± 7.17% relative to WT, *p* = .019 vs. AD + AAV_EGFP_; Figure 1b and c) significantly decreased the levels of APP (AD + AAV_TRPV1_: 249.05 ± 16.66% relative to WT, *p* = .041 vs. AD + AAV_EGFP_; Figure 1b and d), c99 (AD + AAV_TRPV1_: 299.52 ± 44.78% relative to WT, *p* = .021 vs. WT, *p* = .042 vs. AD + AAV_EGFP_; Figure 1b and e), BACE1 (AD + AAV_TRPV1_: 112.86 ± 10.89% relative to WT, *p* = .014 vs. AD + AAV_EGFP_; Figure 1b and f), and PS1 (AD + AAV_TRPV1_: 103.23 ± 15.26% relative to WT, *p* = .049 vs. AD + AAV_EGFP_; Figure 1b and f). Notably, downregulation of TRPV1 (AD + AAV_shTRPV1_: 24.83 ± 8.91% relative to WT, *p* = .025 vs. AD + AAV_EGFP_; Figure 1b and c) had no effects on APP (AD + AAV_shTRPV1_: 366.09 ± 50.76% relative to WT, *p* = .711 vs. AD + AAV_EGFP_; Figure 1b and d) and PS1 (AD + AAV_shTRPV1_: 167.98 ± 18.52% relative to WT, *p* = .437 vs. AD + AAV_EGFP_; Figure 1b and h), but significantly increased the expression of c99 (AD + AAV_shTRPV1_:759.34 ± 80.02% relative to WT, *p* < .001 vs. WT, *p* = .002 vs. AD + AAV_EGFP_; Figure 1b and e) and BACE1 (AD + AAV_shTRPV1_: 165.25 ± 4.95% relative to WT, *p* = .049 vs. AD + AAV_EGFP_; Figure 1b and g). Neither AAV_TRPV1_ nor AAV_shTRPV1_ affected the expression of c89 (AD + AAV_TRPV1_: 431.13 ± 50.89% relative to WT, *p* = .258 vs. AD + AAV_EGFP;_ AD + AAV_shTRPV1_: 835.83 ± 189.97% relative to WT, *p* = .190 vs. AD + AAV_EGFP_; Figure 1b and f).

The neuritic plaques formed by Aβ accumulation are a pathological hallmark of AD. Our results have shown that upregulation of TRPV1 alleviates the APP processing. We therefore wanted to determine the effect of TRPV1 on the formation of neuritic plaques in AD model mice. The results showed that upregulation of TRPV1 obviously decreased the number of neuritic plaques in the hippocampus of APP23/PS45 mice (WT: *n* = 14 slices from 3 mice; AD + AAV_EGFP_: *n* = 34 slices from 8 mice, 36.69 ± 4.13, *p* < .001 vs. WT; AD + AAV_TRPV1_: *n* = 35 slices from 9 mice, 16.63 ± 2.78, *p* < .001 vs. AD + AAV_EGFP_; Figure 1g and h), whereas downregulation of TRPV1 had no effect on the number of neuritic plaques (AD + AAV_shTRPV1_: *n* = 42 slices from 9 mice, 28.60 ± 3.11, *p* = .086 vs. AD + AAV_EGFP_; Figure 1g and h). These results suggest that TRPV1 downregulates APP processing and Aβ deposition.

### 2.2 TRPV1 rescues hippocampal CA1 LTP in APP23/PS45 mice

**Figure 2:**
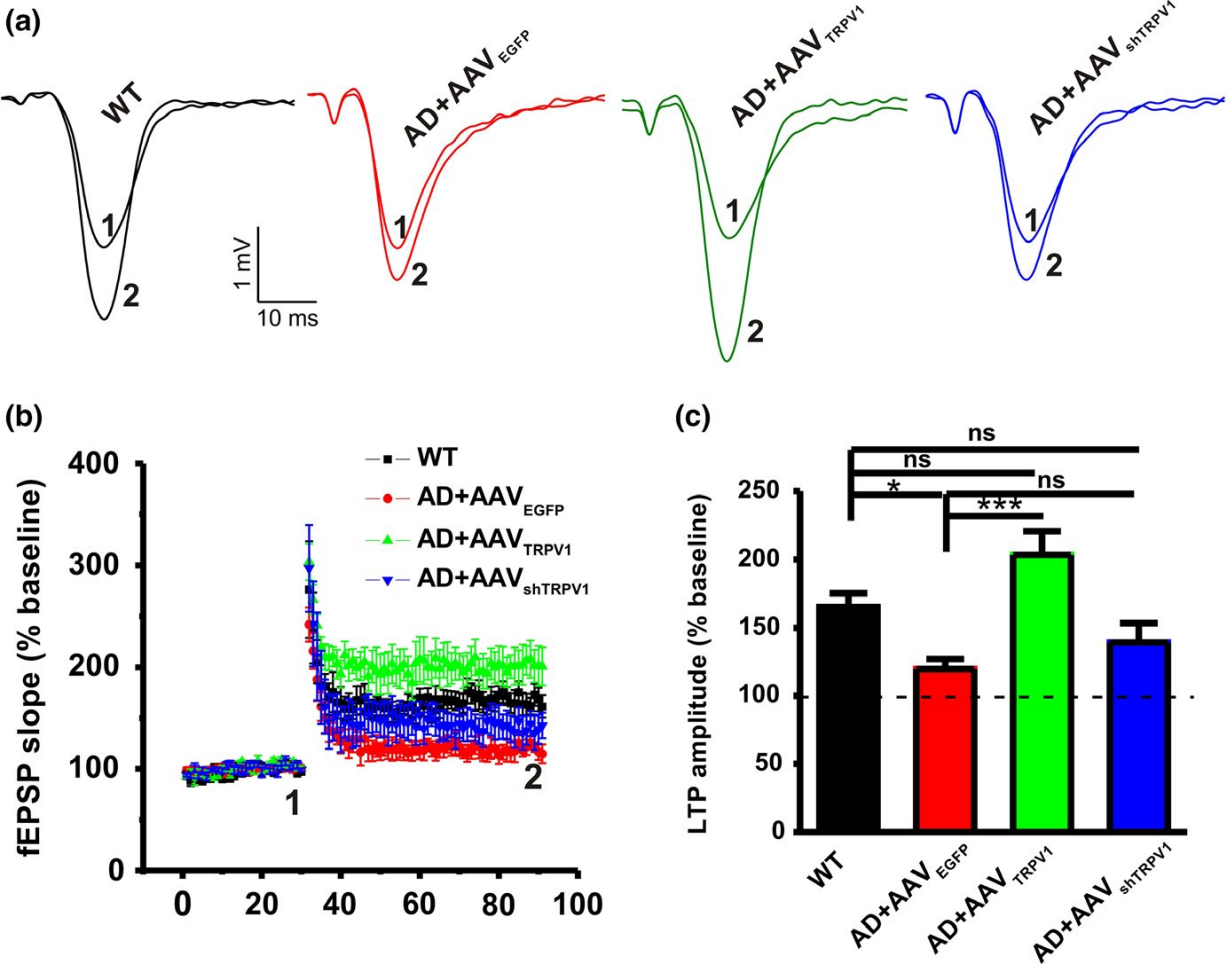
TRPV1 rescues the impairment of LTP in the CA1 area of hippocampus in APP23/PS45 mice. Representative fEPSP traces (a) and plots of the normalized slopes (b) of the fEPSP 5 min before and 55 min after TBS delivery. (c) Bar graphs of the average percentage changes in the fEPSP slope 55–60 min after TBS delivery (*n* = 5–6 slices from 4–6 mice in each group). One‐way ANOVA: *F*_(3,19)_ = 7.330, *p* = .002. Data are expressed as means ± *SEM*, *p < .05, ***p < .001

Hippocampal LTP has been considered as a cellular mechanism underlying learning and memory (Bliss & Collingridge, 1993; Collingridge et al., 2004; Malenka & Nicoll, 1999). Therefore, we further detected the influence of TRPV1 on hippocampal LTP in AD model mice. Our results showed that a reliable hippocampal CA1 LTP was induced in WT mice (WT: *n* = 5 slices from 5 mice, 165.30 ± 100% baseline; Figure 2). However, the LTP was apparently impaired in APP23/PS45 mice (AD + AAV_EGFP_: *n* = 5 slices from 4 mice, 119.71 ± 3.36% baseline, *p* = .036 vs. WT; Figure 2). Upregulation of TRPV1 could reverse the LTP impairment (AD + AAV_TRPV1_: *n* = 6 slices from 4 mice, 203.46 ± 17.22% baseline, *p* < .001 vs. AD + AAV_EGFP_; Figure 2), whereas AAV_shTRPV1_ treatment had no effect on LTP induction (AD + AAV_shTRPV1_: *n* = 6 slices from 4 mice, 139.17 ± 14.29% baseline, *p* = .324 vs. AD + AAV_EGFP_; Figure 2) in APP23/PS45 mice. Notably, AAV microinjection did not affect basal synaptic transmission because the input–output curve remained unchanged among these groups (Figure S1). Together, these results indicate that TRPV1 could rescue the hippocampal CA1 LTP impairment in AD model mice.

### 2.3 TRPV1 inhibits AMPAR endocytosis by interacting with GluA2 subunit

**Figure 3:**
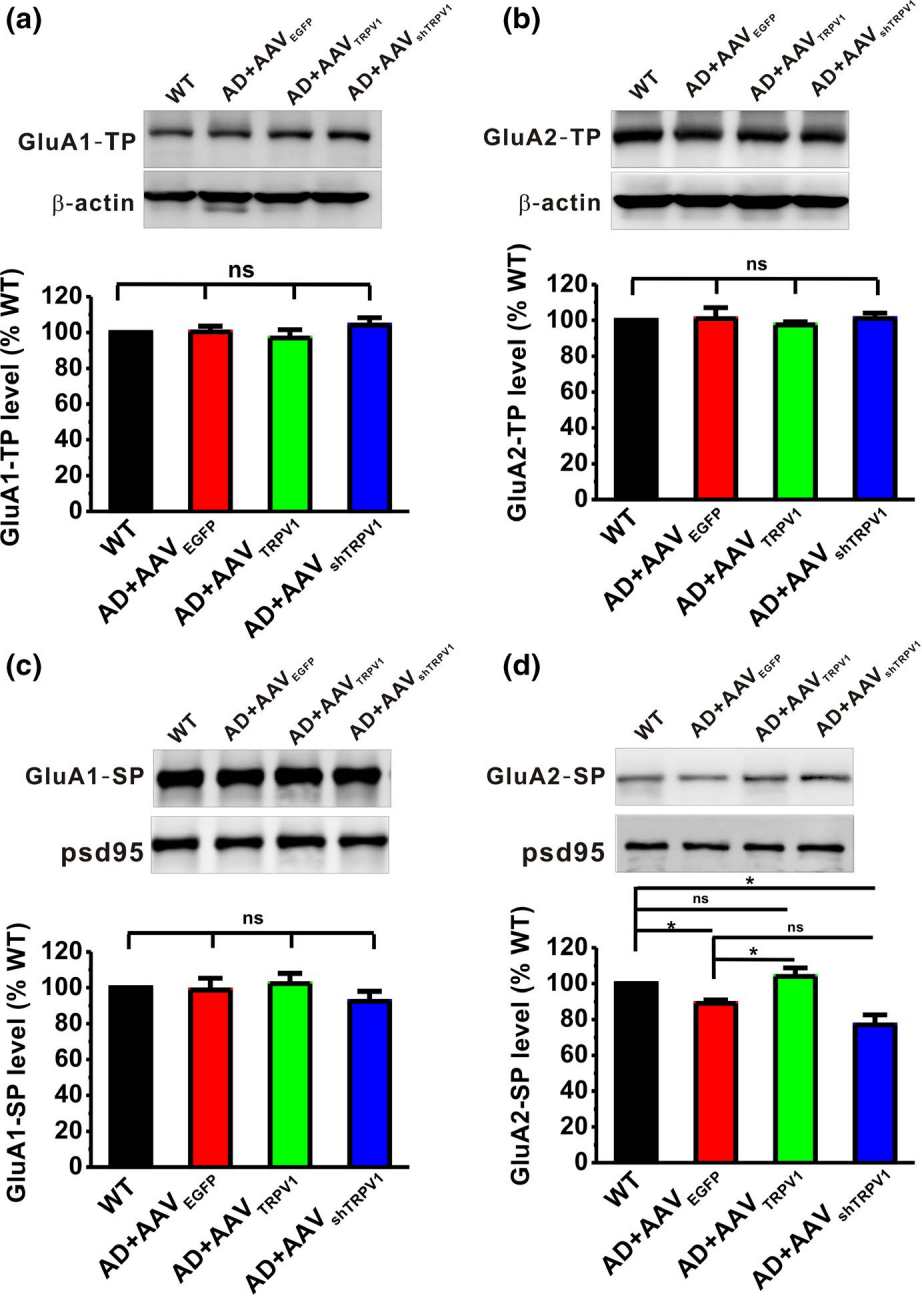
TRPV1 increases the expression of GluA2 in the synapse. The relative protein levels of total GluA1 (a), total GluA2 (b), synaptic GluA1(c), and synaptic GluA2 (d) are normalized by WT mice (*n* = 4–6 in each group). One‐way ANOVA: *F*_(3,21)_ = 0.707, *p* = .559 for total GluA1; *F*_(3,13)_ = 0.351, *p* = .789 for total GluA2; *F*_(3,21)_ = 0.636, *p* = .601 for synaptic GluA1; and *F*_(3,13)_ = 10.829, *p* = .001 for synaptic GluA2. Data are expressed as mean ± *SEM*, *p < .05

Electrophysiological data have revealed that TRPV1 is able to rescue the hippocampal CA1 LTP in the APP23/PS45 mice, while our recent study has reported that AMPAR endocytosis plays critical role in medicating LTP decay (Dong et al., 2015). In order to detect whether TRPV1 can reverse LTP impairment by affecting AMPAR endocytosis, we next examined the expression of AMPARs including GluA1 and GluA2 subunits in the total lysate and synaptic fraction. The results showed that either upregulation or knockdown of TRPV1 did not affect the expression of total GluA1 (GluA1‐TP; Figure 3a) and GluA2 (GluA2‐TP; Figure 3b). However, the synaptic protein level of GluA2 (GluA2‐SP) was significantly decreased in APP23/PS45 mice (AD + AAV_EGFP_: 88.02 ± 1.46% relative to WT, *p* = .043 vs. WT; Figure 3d) compared to the WT group. More importantly, upregulation of TRPV1 restored the expression of synaptic GluA2 (AD + AAV_TRPV1_: 104.10 ± 4.60% relative to WT, *p* = .453 vs. WT, *p* = .010 vs. AD + AAV_EGFP;_ Figure 3d), whereas AAV_shTRPV1_ had no effect on the synaptic GluA2 expression (AD + AAV_shTRPV1_:76.89 ± 5.72% relative to WT, *p* = .001 vs. WT, *p* = .057 vs. AD + AAV_EGFP;_ Figure 3d). Notably, there was no significant difference in the expression level of synaptic GluA1 among these four groups (Figure 3c).

**Figure 4:**
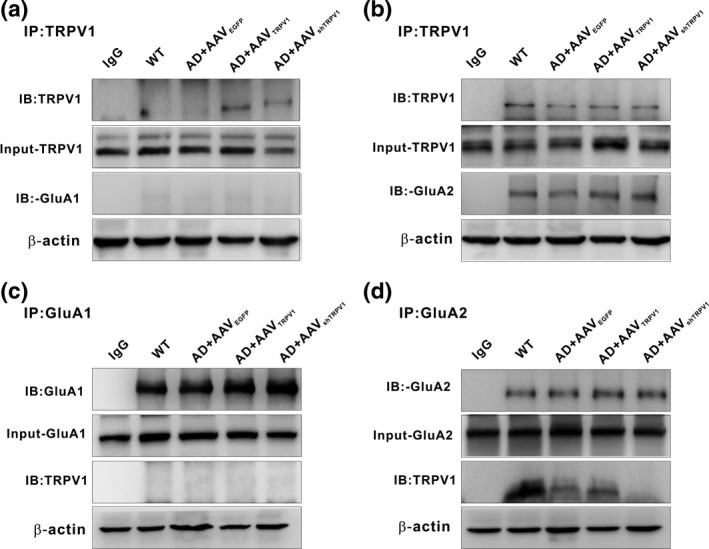
TRPV1 interacts with GluA2 subunit of AMPAR. (a and b) Immunoprecipitation (IP) of hippocampal samples from WT and APP23/PS45 mice after extraction in lysis buffer with antibodies to TRPV1 or negative control antibodies (IgG) and immunoblot analysis with the indicated antibodies to the TRPV1, GluA1 (a), and GluA2 (b). (c and d) IP of hippocampal samples from WT and APP23/PS45 mice after extraction in lysis buffer with antibodies to GluA1, GluA2, or IgG and immunoblot analysis with the indicated antibodies to the TRPV1, GluA1 (c), and GluA2 (d)

To determine whether TRPV1 physically associates with AMPARs, and subsequently resulting in AMPAR endocytosis, we next performed co‐immunoprecipitation experiments in extracts of brain samples from WT and APP23/PS45 mice. The results demonstrated that TRPV1 co‐immunoprecipitated with GluA2 (Figure 4b), but not GluA1 (Figure 4a). To further confirm the finding that TRPV1 only interacted with GluA2, we next used antibodies to GluA1 and GluA2 to precipitate TRPV1 and got the similar results that only GluA2 (Figure 4d), but not GluA1 (Figure 4c), was able to co‐immunoprecipitate with TRPV1. More importantly, the interaction of TRPV1 with GluA2 was reduced in APP23/PS45 mice compared to WT, and upregulation of TRPV1 increased the interaction between GluA2 and TRPV1 (Figure 4d). Collectively, these data suggest that expression of TRPV1 regulates AMPAR endocytosis through interaction with the GluA2 subunit.

### 2.4 Genetic upregulation of TRPV1 rescues memory decline in APP23/PS45 mice

**Figure 5:**
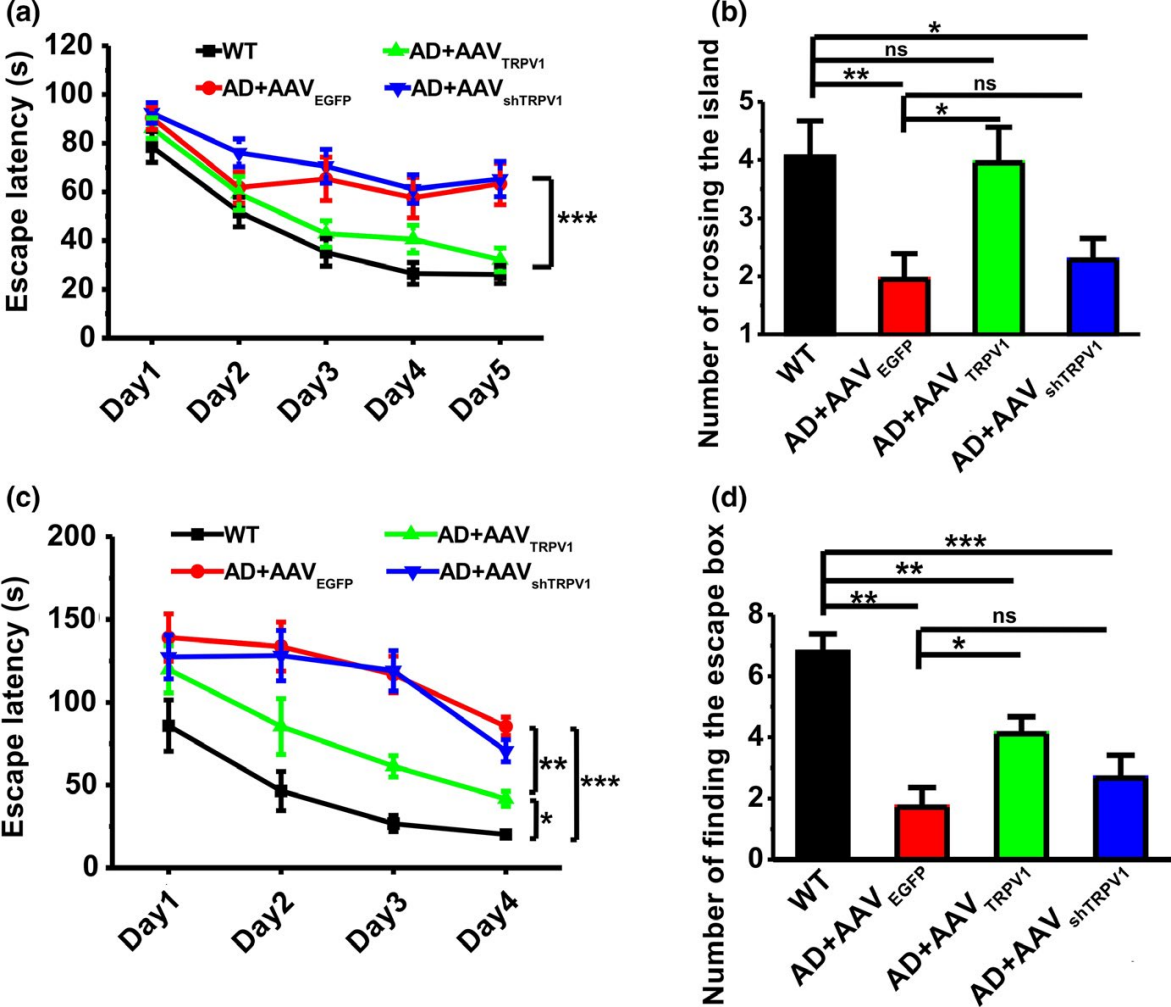
Genetic upregulation of TRPV1 rescues learning and memory deficits in APP23/PS45 mice. (a) The escape latency to the hidden platform during spatial learning in the Morris water maze paradigm (*n* = 16–24 in each group). Repeated measures ANOVA: *F*_(3,74)_ = 9.748, *p* < .001. (b) The number of entries into the platform zone. One‐way ANOVA: *F*_(3,74)_ = 3.281, *p* = .026. (c) The latency to the escape box during spatial learning in the Barnes maze paradigm (*n* = 8–9 in each group). Repeated measures ANOVA: *F*_(3,41)_ = 20.139, *p* < .001. (d) The occurrence of head dips to the escape hole during memory retrieval. One‐way ANOVA: *F*_(3,41)_ = 10.793, *p* < .001. Data are expressed as mean ± *SEM*, *p < .05, **p < .01, ***p < .001

Aforementioned results have revealed that TRPV1 can reduce AD‐related neuropathologies in APP23/PS45 mice. To directly examine whether TRPV1 could alleviate cognitive impairments in these AD model mice, the Morris water maze test was introduced to measure the spatial learning and memory. During the Morris water maze training, the escape latency in APP23/PS45 group (AD + AAV_EGFP_, *n* = 18) was much longer than those in the WT group (WT, *n* = 16) (Day 1:77.72 ± 6.14 s for WT, 89.17 ± 4.98 s for AD + AAV_EGFP_; Day 2:45.66 ± 6.36 s for WT, 63.48 ± 6.48 s for AD + AAV_EGFP_; Day 3:31.59 ± 4.35 s for WT, 67.86 ± 8.97 s for AD + AAV_EGFP_; Day 4:24.41 ± 3.17 s for WT, 59.78 ± 8.44 s for AD + AAV_EGFP_; Day 5:25.94 ± 2.89 s for WT, 63.41 ± 9.02 s for AD + AAV_EGFP_; *p* < .001 vs. WT; Figure 5a). AAV_TRPV1_ (AD + AAV_TRPV1_, *n* = 19) treatment significantly shortened the escape latency (Day 1:84.36 ± 4.56 s; Day 2:57.01 ± 7.14 s; Day 3:42.89 ± 5.74 s; Day 4:35.84 ± 5.45 s; Day 5:28.94 ± 4.44 s; *p* = .003 vs. AD + AAV_EGFP_; Figure 5a) compared to those in AD group, whereas AAV_shTRPV1_ (AD + AAV_shTRPV1_, *n* = 24) treatment displayed no difference with the AD group (Day 1:89.23 ± 4.09 s; Day 2:70.82 ± 5.99 s; Day 3:68.52 ± 6.58 s; Day 4:54.49 ± 5.77 s; Day 5:59.29 ± 7.23 s; *p* = .964 vs. AD + AAV_EGFP_; Figure 5a). Twenty‐four hours after the last training trial, a probe test with the platform removed was performed to examine long‐term spatial memory retrieval. The results revealed that spatial memory retrieval dramatically impaired in AD mice since the number of entries into the platform zone was reduced (WT: 4.13 ± 0.71; AD + AAV_EGFP_: 2.00 ± 0.46, *p* = .008 vs. WT; Figure 5b). AAV_TRPV1_ treatment significantly increased the number of entries into the platform zone (AD + AAV_TRPV1_: 3.68 ± 0.54, *p* = .571 vs. WT, *p* = .028 vs. AD + AAV_EGFP_; Figure 5b), whereas AAV_shTRPV1_ treatment displayed no difference compared with the AD group (AD + AAV_shTRPV1_: 2.58 ± 0.39, *p* = .040 vs. WT, *p* = .415 vs. AD + AAV_EGFP_; Figure 5b).

To further evaluate the effect of TRPV1 on amelioration of learning and memory in AD model mice, we next performed another hippocampus‐dependent learning and memory task, the Barnes maze test. During spatial learning period, the escape latency for finding the escape box in APP23/PS45 mice (AD + AAV_EGFP_, *n* = 8) was much longer than those in WT mice (WT, *n* = 9) (Day 1:85.95 ± 15.50 s for WT, 139.07 ± 14.27 s for AD + AAV_EGFP_; Day 2:46.45 ± 11.88 s for WT, 133.75 ± 14.78 s for AD + AAV_EGFP_; Day 3:26.72 ± 4.87 s for WT, 116.93 ± 10.99 s for AD + AAV_EGFP_; Day 4:20.12 ± 1.34 s for WT, 85.48 ± 5.59 s for AD + AAV_EGFP_; *p* < .001 vs. WT; Figure 5c). AAV_TRPV1_ (AD + AAV_TRPV1_, *n* = 9) treatment significantly shortened escape latency for searching for the escape box (Day 1:119.95 ± 14.28 s; Day 2:5.37 ± 16.93 s; Day 3:61.31 ± 6.42 s; Day 4:41.70 ± 4.58 s; *p* = .001 vs. AD + AAV_EGFP_; Figure 5c), whereas AAV_shTRPV1_ treatment (AD + AAV_shTRPV1_, *n* = 9) displayed no difference compared with the AD group (Day 1:127.42 ± 13.16 s; Day 2:128.24 ± 15.16 s; Day 3:119.11 ± 12.22 s; Day 4:70.66 ± 6.70 s; *p* = .499 vs. AD + AAV_EGFP_; Figure 5c). Twenty‐four hours after the last training trial, a long‐term spatial memory retrieval test was performed with the escape box blocked. The results showed that memory retrieval was impaired in AD model mice since the number of finding the escape box was significantly decreased compared to WT control (WT: 6.78 ± 0.60; AD + AAV_EGFP_: 1.71 ± 0.64; *p* < .001 vs. WT; Figure 5d). AAV_TRPV1_ treatment increased the number of finding the escape box (AD + AAV_TRPV1_: 4.11 ± 0.56, *p* = .006 vs. WT, *p* = .030 vs. AD + AAV_EGFP;_ Figure 5d), whereas AAV_shTRPV1_ treatment displayed no difference compared to AD group (AD + AAV_shTRPV1_: 2.67 ± 0.75, *p* < .001 vs. WT, *p* = .477 vs. AD + AAV_EGFP_; Figure 5d). Besides learning and memory deficits, patients with AD are often accompanied by depression, and laboratory research has shown that TRPV1 activation may affect anxiogenic responses and depression‐related behaviors (Abdelhamid, Kovacs, Nunez, & Larson, 2014; Kasckow, Mulchahey, & Geracioti, 2004). We here further found that TRPV1 upregulation by AAV_TRPV1_ could significantly ameliorate anxiety‐ and depression‐like behaviors in APP23/PS45 mice during the elevated plus maze (Figure 2a and b), three‐chambered social interaction (Figure 2c), and force swimming tests (Figure 2d). Collectively, these results indicate that genetic upregulation of TRPV1 by AAV microinjection succeeds in rescuing the decline of cognitive and emotional functions in AD model mice.

### 2.5 Pharmacological activation of TRPV1 rescues memory decline in APP23/PS45 mice

**Figure 6:**
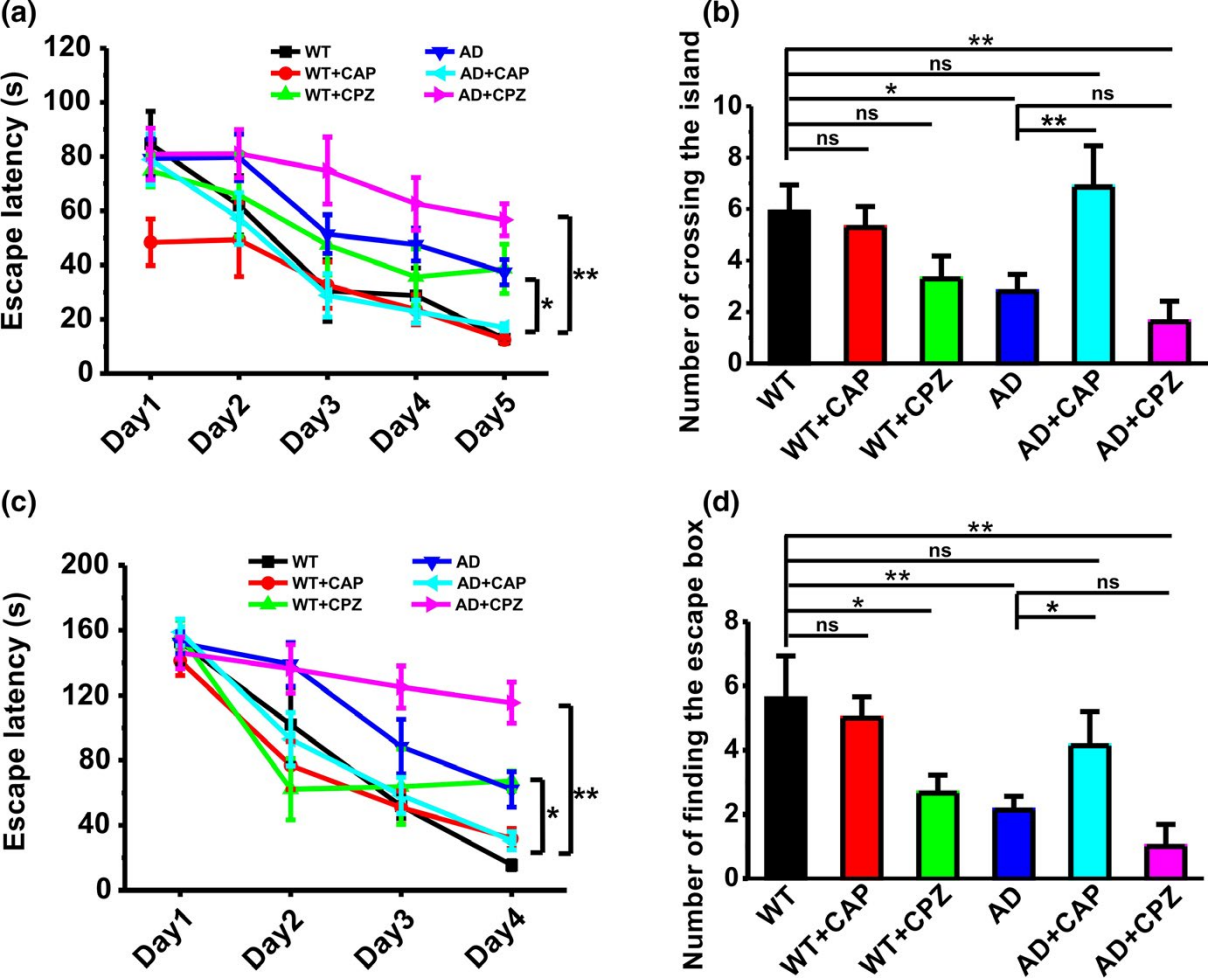
Capsaicin rescues learning and memory deficits in APP23/PS45 mice. (a) The escape latency to the hidden platform during spatial learning in the Morris water maze paradigm (*n* = 7–10 in each group). Repeated measures ANOVA: *F*_(5,42)_ = 6.274, *p* < .001. (b) The number of entries into the platform zone. One‐way ANOVA: *F*_(5,42)_ = 4.276, *p* = .003. (c) The latency to the escape box during spatial learning in the Barnes maze paradigm (*n* = 5–8 in each group). Repeated measures ANOVA: *F*_(5,37)_ = 8.805, *p* < .001. (d) The occurrence of head dips to the escape hole during memory retrieval. One‐way ANOVA: *F*_(5,37)_ = 5.024, *p* = .001. Data are expressed as mean ± *SEM*, *p < .05, **p < .01

As genetic upregulation of TRPV1 is difficult to be used in the clinical treatment, we next wanted to determine whether pharmacological activation of TRPV1 by its agonist capsaicin (CAP, 1 mg/kg, i.p.) is able to ameliorate the spatial learning and memory deficits in AD model mice. The Morris water maze and Barnes maze tests were used to measure the spatial learning and memory in mice treated with CAP or TRPV1 antagonist capsazepine (CPZ, 1 mg/kg, i.p.). The results showed that CAP treatment (WT + CAP: *n* = 7; 48.42 ± 8.62 s on Day 1, 49.39 ± 13.65 s on Day 2, 32.61 ± 8.63 s on Day 3, 23.54 ± 5.58 s on Day 4, 12.46 ± 1.42 s on Day 5; *p* = .191 vs. WT; Figure 6a) had no effect on spatial learning during the Morris water maze test in WT mice (WT: *n* = 8; 84.67 ± 12.05 s on Day 1, 62.01 ± 10.98 s on Day 2, 30.58 ± 11.32 s on Day 3, 28.78 ± 10.17 s on Day 4, 12.74 ± 1.50 s on Day 5; Figure 6a), as reflected by taking similar time to find the hidden platform. However, the escape latency for searching for the hidden platform in APP23/PS45 mice treated with CAP was significantly shortened (AD + CAP: *n* = 7; 78.89 ± 9.34 s on Day 1, 57.20 ± 9.58 s on Day 2, 28.85 ± 7.98 s on Day 3, 22.80 ± 4.28 s on Day 4, 17.03 ± 1.39 s on Day 5; *p* = .020 vs. AD; Figure 6a), compared to vehicle treatment (AD: *n* = 10; 79.24 ± 7.22 s on Day 1, 79.62 ± 8.59 s on Day 2, 51.43 ± 7.09 s on Day 3, 47.58 ± 6.05 s on Day 4, 37.34 ± 4.66 s on Day 5; *p* = .040 vs. WT; Figure 6a). Notably, TRPV1 antagonist capsazepine (CPZ, 1 mg/kg, i.p.) administration did not affect the escape latency for searching for the hidden platform in both WT (WT + CPZ: *n* = 7; 74.71 ± 5.84 s on Day 1, 65.80 ± 15.18 s on Day 2, 47.48 ± 11.07 s on Day 3, 35.58 ± 10.42 s on Day 4, 38.67 ± 9.09 s on Day 5; *p* = .275 vs. WT; Figure 6a) and APP23/PS45 mice (AD + CPZ: *n* = 8; 80.87 ± 9.56 s on Day 1, 81.06 ± 8.89 s on Day 2, 74.81 ± 12.38 s on Day 3, 62.55 ± 9.71 s on Day 4, 56.66 ± 5.91 s on Day 5; *p* = .100 vs. AD; Figure 6a), compared to vehicle treatment. The results from long‐term spatial memory retrieval test revealed that treatment with CAP (AD + CAP: 6.86 ± 1.61, *p* = .490 vs. WT, *p* = .004 vs. AD; Figure 6b), but not CPZ (AD + CPZ: 1.63 ± 0.80 s, *p* = .003 vs. WT, *p* = .368 vs. AD; Figure 6b), reversed the memory decline in APP23/PS45 mice since the number of entries into the platform zone was dramatically increased, compared to vehicle treatment.

Next, we also evaluate the effects of CAP on learning and memory in APP23/PS45 mice in the paradigm of Barnes maze performance. The escape latency for searching for the escape box in APP23/PS45 mice (AD: *n* = 8; 152.21 ± 6.66 s on Day 1, 139.01 ± 13.53 s on Day 2, 88.49 ± 16.78 s on Day 3, 62.06 ± 10.95 s on Day 4; *p* = .015 vs. WT; Figure 6c) was much longer than those in WT (WT: *n* = 5; 152.81 ± 13.99 s on Day 1, 102.02 ± 22.79 s on Day 2, 51.86 ± 7.60 s on Day 3, 15.47 ± 2.69 s on Day 4; Figure 6c). CAP treatment (AD + CAP: *n* = 7; 158.75 ± 8.24 s on Day 1, 93.00 ± 16.43 s on Day 2, 58.49 ± 11.06 s on Day 3, 30.53 ± 5.54 s on Day 4; *p* = .023 vs. AD; Figure 6c) significantly shortened escape latency for searching for the escape box, whereas CPZ did not affect the escape latency (AD + CPZ: *n* = 7; 146.03 ± 9.65 s on Day 1, 136.32 ± 14.92 s on Day 2, 125.04 ± 12.96 s on Day 3, 115.42 ± 12.61 s on Day 4; *p* = .065 vs. AD; Figure 6c). The results from long‐term spatial memory retrieval test showed that CAP (AD + CAP: 4.14 ± 1.06, *p* = .237 vs. WT, *p* = .043 vs. AD; Figure 6d), but not CPZ (AD + CPZ: 1.00 ± 0.69, *p* = .001 vs. WT, *p* = .301 vs. AD; Figure 6d), reversed the memory deficits in APP23/PS45 mice, as reflected by much more times to finding the escape box. Taken together, these findings indicate that pharmacological activation of TRPV1 by CAP significantly reverses the memory decline in AD model mice.

## 3 DISCUSSION

In the present study, we find that TRPV1 expression is significantly reduced in the brains of APP23/PS45 transgenic model mice of AD. We also report that upregulation of TRPV1 alleviates AD‐related neuropathologies and inhibits AMPAR endocytosis via interacting with GluA2 subunit, which may subsequently contribute to the amelioration of cognitive function and synaptic plasticity. Meanwhile, TRPV1 activation by CAP could also improve the learning and memory in APP23/PS45 double transgenic mice. Collectively, the current study demonstrates the protective effect of TRPV1 on AD pathogenesis, suggesting that it may serve as a potential therapeutic molecule for AD.

Progressive cognitive decline is the main clinical symptom in AD, and synaptic plasticity is generally accepted as a critical phenomenon used by the brain for adapting or learning from experiences in our environment (Bliss & Collingridge, 1993; Collingridge et al., 2004; Malenka & Nicoll, 1999). It has recently been demonstrated that TRPV1 channels are widely expressed in the central nervous system and participate in long‐term synaptic plasticity in the CA1 region (Hurtado‐Zavala et al., 2017; Li et al., 2008; Marsch et al., 2007; Mezey et al., 2000; Toth et al., 2005). Thus, regulation of TRPV1 may alleviate synaptic and cognitive impairments during AD development. Indeed, our recent study has demonstrated that TRPV1 activation by CAP could improve the synaptic and cognitive functions in Aβ‐induced mouse model of AD (Chen et al., 2017). Jiang and colleagues have also reported that activation of TRPV1 can mitigate stress‐induced AD‐like neuropathological alterations and cognitive impairment in rats (Jiang et al., 2013). Accordingly, in the present study, we found that upregulation of TRPV1 by AAV microinjection succeeded in ameliorating the synaptic and cognitive functions in APP23/PS45 transgenic mouse model of AD. We also reported that pharmacological activation of TRPV1 by its agonist CAP could rescue the deficits of spatial learning and memory in the APP23/PS45 mice, indicating that activation of TRPV1 may serve as a potential strategy for AD treatment.

To date, despite the fact that all of the clinical trials for AD focused on Aβ have failed, accumulating evidence from laboratories and clinics worldwide supports the view that an imbalance between production and clearance of Aβ and related Aβ peptides is a very early, often initiating factor during the progression of AD (Beyreuther & Masters, 1991; Hardy & Higgins, 1992; Selkoe, 1991). Therefore, Aβ has been generally recognized as the culprit of AD (Wilcock & Griffin, 2013), which was generated from proteolytic cleavages of APP by the β‐ and γ‐secretases (Querfurth & LaFerla, 2010), and reducing the amount of Aβ might be a potential therapeutic strategy for AD. TRPV1 activation causes Ca^2+^ influx, and dysregulation of Ca^2+^ is involved in the pathogenesis of AD. For instance, the levels of calcium activity are increased in AD patients (Johnson et al., 1997) and elevated Ca^2+^ induce BACE1 expression and consequently result in an increase in Aβ production (Cho, Jin, Youn, Huh, & Mook‐Jung, 2008; Mata, 2018) and cell death (Pierrot, Ghisdal, Caumont, & Octave, 2004). However, contradictory results have shown that familial AD‐linked PS1 or PS2 mutation attenuates calcium entry and thus increasing Aβ production (Fedeli, Filadi, Rossi, Mammucari, & Pizzo, 2019; Yoo et al., 2000), whereas constitutive activation of Ca^2+^ entry reduces Aβ secretion (Zeiger et al., 2013). Thus, genetic or pharmacological activation of TRPV1 may increase Ca^2+^ influx, reduce APP processing, and consequently improve synaptic plasticity and memory in APP23/PS45 model mice of AD.

It has been well documented that Aβ accumulation could dramatically interfere with synaptic transmission, leading to impairment of LTP (Nalbantoglu et al., 1997; Shankar et al., 2008), and the GluA2‐dependent AMPAR endocytosis contributes to the decay of LTP, thereby impairing learning and memory (Dong et al., 2015). However, the mechanisms by which induced AMPAR endocytosis are still unclear. Here, we reported that AMPAR endocytosis was increased in APP23/PS45 transgenic mice, and upregulation of TRPV1 by AAV microinjection significantly reduced AMPAR endocytosis. Importantly, TRPV1 was able to interact with GluA2, but not GluA1, and their interactions were reduced in APP23/PS45 transgenic mice. Upregulation of TRPV1 may prolong activation of AMPAR in the hippocampus due to enhanced Ca^2+^ influx following TRPV1 activation, and thereby inhibiting GluA2‐dependent AMPAR endocytosis. Notably, how TRPV1 interacts with GluA2 remains to be further detected.

In summary, our study demonstrates that genetic or pharmacological activation of TRPV1 not only improves hippocampal LTP and memory but also reduces neuritic plaques in mouse model of AD, suggesting a potential therapeutic role of TRPV1 for learning and memory deficits associated with both patients with AD and aged populations.

## 4 EXPERIMENTAL PROCEDURES

### 4.1 Animals

APP23/PS45 transgenic mice were maintained at Children's Hospital of Chongqing Medical University Animal Care Centre. Mice were housed under 12‐hr light and 12‐hr dark cycle (lights on from 7:00 a.m. to 7:00 p.m.) with free access to food and water in a temperature and humidity controlled SPF room. The genotype of the mice was confirmed by PCR using DNA from tail tissues. All animal experiments were conducted in accordance with the Chongqing Science and Technology Commission guidelines and approved by the Chongqing Medical University Animal Care Committee.

### 4.2 Drugs and treatment

TRPV1 agonist capsaicin (CAP) and antagonist capsazepine (CPZ) were purchased from Sigma‐Aldrich, which were dissolved in a 1:1:8 mixture of Tween 80: ethanol: saline. Mice received drug injection daily from the age of 1.5 months to the end of the behavioral test.

### 4.3 Adeno‐associated virus and microinjection

To overexpress or knockdown TRPV1 in vivo, adeno‐associated virus‐mediated TRPV1 (AAV_TRPV1_) or TRPV1 small hairpin RNA (AAV_shTRPV1_) was constructed by OBiO Technology (Shanghai, China). Titers were 3 × 10^12^ TU/ml. Mice were anesthetized with sodium pentobarbital (60 mg/kg, i.p.), and the core temperature was maintained at 36.5 ± 0.5°C. Atropine (0.4 mg/kg, i.p.) was also given to help relieve respiratory congestion. Scalp skin was shaved with clippers and disinfected using iodine before mice were mounted on a stereotaxic instrument. After opening the scalp skin and exposing the skull, 1 μl of AAV_TRPV1_ or AAV_shTRPV1_ was microinjected into each hippocampal CA1 area by drilled hole (−2.3 mm and −2.5 mm posterior, ±2.0 mm lateral and 2.5 mm ventral relative to bregma). Animals received AAV microinjections at the age of 1.5 months and performed behavioral test at the age of 3 months.

### 4.4 Morris water maze test

The Morris water maze consists of a circular stainless steel pool (150 cm in diameter) filled with opaque white paint as previously described (Dong et al., 2015; Du et al., 2019). The pool was surrounded by light blue curtains, and 3 distal visual cues were fixed to the curtains. A CCD camera was suspended above the pool center to record the animal's swimming path, and video output was digitized by an ANY‐maze tracking system (Stoelting). Twenty‐four hours before spatial training, the animals were allowed to adapt to the maze for a 120‐s free swim. The animals were then trained in the spatial learning task for 4 trials per day for 5 consecutive days. In each trial, mice were placed into water from 4 starting positions (NE, NW, SW, and SE), facing to the pool wall. They were then required to swim to find the hidden platform (7.5 cm in diameter), which was submerged 1 cm below the opaque water surface in a fixed position in the SW quadrant. During each trial, mice were allowed to swim until they found the hidden platform where they remained for 20 s before being returned to home cage. Mice that failed to find the hidden platform in 120 s were guided to the platform where they remained for 20 s. Twenty‐four hours after the final training trial, a probe test was conducted. Mice were returned to the pool from a novel drop point with the hidden platform absent for 120 s, and their swim path was recorded.

### 4.5 Barnes maze test

The apparatus consists of a white circular platform 0.75 m in diameter, with 18 holes (5 cm in diameter) placed at the edges, and with an escape box located underneath one of these holes as previously described (Yu et al., 2018). A CCD camera suspended above the maze center records the latency and count of errors of finding the escape box, and video outputs are digitized by an ANY‐maze video tracking system (Stoelting). Twenty‐four hours before spatial training, the animals were allowed to adapt to the maze for 3 min. The animals were then trained in spatial learning task for 4 trials per day for 4 days, with an inter‐trial interval of 15 min. In each trial, the animal was placed in the center of the maze, and the time travelled to get to the escape box was measured. If the animal found and entered into the box within 3 min, the animal was returned to its home cage; if the animal failed to find the escape box, or if it found the box but did not enter into the box within 3 min, it would be gently guided to the box and held there for 60 s before being returned to the home cage. Twenty‐four hours after the final training trial, mice were returned to the maze and a 3‐min probe test was performed with the escape box blocked.

### 4.6 Electrophysiology in vitro

Mice (4 months old) were deeply anesthetized with urethane (1.5 g/kg, i.p.) and transcardially perfused with artificial cerebral spinal fluid (ACSF) (in mM: NaCl 124, KCl 2.8, NaH_2_PO_4_.H_2_O 1.25, CaCl_2_ 2.0, MgSO_4_ 1.2, Na‐vitamin C 0.4, NaHCO_3_ 26, Na‐lactate 2.0, Na‐pyruvate 2.0 and D‐glucose 10.0, pH = 7.4) prior to decapitation as described previously (Du et al., 2019; Huang et al., 2017). Then, hippocampal slices were coronally sectioned (400 μm) with a vibratome (VT1200S, Leica Microsystems, Bannockburn, IL) with 95% O_2_ and 5% CO_2_, and then were incubated in ACSF for 2 hr at 35°C. A bipolar stimulating electrode was placed at the Schaffer collaterals of dorsal hippocampus CA3 pyramidal neurons, and a recording pipette filled with ASCF was placed at the ipsilateral striatum radiatum of the hippocampal CA1 area. An input–output curve was measured at different current stimulation from 0.05 to 0.4 mA, and test EPSPs were evoked at a frequency of 0.05 Hz and at a stimulus intensity adjusted to around 50% of the maximal response size. After a 30‐min stable baseline, theta burst stimulation (TBS) was given to induce LTP. TBS consisted of 2 trains of stimuli (at 20 s interval), with each train composed of 5 bursts (4 pulses at 100 Hz in each burst) at an inter‐burst interval of 200 ms. Data acquisition was performed with the PatchMaster v2.73 software (HEKA Elektronik, Lambrecht/Pfalz, Germany).

### 4.7 Immunohistochemistry staining

Mice were euthanized with urethane (3 g/kg, i.p., Sigma) after behavioral testing, and one‐half of the brain was immediately frozen for protein or RNA extraction. The other half of the brain was postfixed in freshly 4% paraformaldehyde (PFA) in 0.1 M phosphate‐buffered saline (PBS, pH 7.4) for 24 hr. Then, the brain was dehydrated with 30% sucrose until it sank to the bottom and serially cut into 30 μm thick coronal sections using Leica Instrument. The sections were incubation with 3% H_2_O_2_ to remove residual peroxidase activity for 30 min. Then, slices were blocked with 10% BSA and incubated with mouse monoclonal 4G8 antibody (1:500) overnight at 4°C. Sections were mounted onto slides, and plaques were visualized by the ABC and DAB method and counted by microscopy at ×40 magnification. The mean plaque count per slice was recorded for each mouse as described previously (Dong et al., 2015; Du et al., 2019).

### 4.8 Western blot assay

After behavioral testing, the hippocampus and cerebral cortex were dissected and weighed immediately and then homogenized in homogeneous buffer in a mortar and pestle. The homogenates were centrifuged (4°C, 12,000 *g*, 15 min) to collect the supernatants. The protein concentration was measured with a BCA protein assay reagent (Thermo Fisher Scientific, Waltham, MA, USA) according to the manufacturer's instruction. Uniform amount of each protein samples (30 μg) was boiled with 5 × loading buffer at 95°C for 10 min. The samples were then separated on 10% SDS‐PAGE and transferred onto an immobilon‐PTM polyvinylidene fluoride (PVDF) membrane. To block nonspecific background, the membranes were incubated with 5% nonfat milk in Tris‐buffered saline containing 0.1% Tween‐20 (TBST) at 37°C for 1 hr. The target proteins were immunoblotted with primary antibody overnight at 4°C to APP (obtained from Professor Weihong Song), BACE1 (1:2000; CST, USA), PS1 (1:3,000; Abcam, USA), TRPV1 (1:200; Millipore, USA), GluA1 (1:500; Abcam, USA), and GluA2 (1:1,000; Abcam, USA). After incubation with goat anti‐rabbit IgG (1:3,000; Abcam, USA) at 37°C for 1 hr, the protein was visualized in the Bio‐Rad Imager using ECL Western blotting substrate (Pierce). β‐actin was the internal reference. The band intensity of each protein was quantified by the Bio‐Rad Quantity One software as described previously (Dong et al., 2015; Du et al., 2019).

### 4.9 Co‐immunoprecipitation

Cerebral hippocampus was homogenized in ice‐cold Co‐IP lysis buffer and proteinase inhibitor mixture. After clearing debris by centrifuge at 12,000 *g* at 4°C, protein concentration in the extracts was determined by BCA assay. The hippocampal samples (500 μg) were incubated with nonspecific lgG or polyclonal rabbit anti‐Glu A1 or anti‐Glu A2 overnight at 4°C, followed by the addition of 40 μl of protein G for 3 hr at 4°C. The precipitate was washed four times with lysis buffer and denatured with SDS sample buffer and separated by 10% SDS‐PAGE.

### 4.10 Statistical analysis

All data are expressed as mean ± *SEM*. ANOVA or two‐tailed Student's *t* tests were used to analyze the data by where appropriate. The significance level was set at p＜0.05.

## CONFLICT OF INTEREST

The authors have declared that no conflict of interest exists.

## AUTHOR CONTRIBUTIONS

YD, MF, and XT performed behavioral and biochemical experiments. ZH and JL performed electrophysiological experiments. YD, JL, and YP analyzed the data. ZD designed the research study and contributed essential reagents or tools. YD and ZD wrote the manuscript. WS and YTW provided helpful discussion.

## Data Availability

The data that support the findings of this study are available from the corresponding author upon reasonable request.
