# Supplementary material for: TRPV1 activation alleviates cognitive and synaptic plasticity impairments through inhibiting AMPAR endocytosis in APP23/PS45 mouse model of Alzheimer’s disease
Source: Aging Cell. 2020 Feb 14;19(3):e13113. doi: 10.1111/acel.13113 (PMC7059138; doi:10.1111/acel.13113)
Supplement: Supplementary file 1 [file ACEL-19-e13113-s001.docx]

**Supplementary Materials**

**Supplementary methods**

**Elevated plus maze**

The elevated plus maze apparatus consist of two opposing open arms (30×5×0.3 cm) and two opposing closed arms (30×5×15 cm). The maze was placed at the height of 75 cm from floor. The mice were individually placed in the middle of the maze facing the open arm for 10 min. The time spent in each arm and total number of entries into each arm were recorded and analyzed by using AnyMaze video tracking software (Stoelting, USA).

**Social interaction test**

The social interaction test was performed in a three-chambered apparatus, which was made from white Plexiglas and contained three chambers with the same dimensions (length × width × height = 20 × 40 × 20 cm for each). An identical cage was used to enclose a stranger mouse or object in each side chamber, and the central chamber was empty. Animal used as “stranger” was a C57BL/6 mouse of the same gender with the same age and no previous contact with the test mice. Test mice were individually acclimated for 10 min into the empty apparatus on the day before the experiment. On the test day, the experimental mouse was placed in the middle chamber that was open to both sides and allowed to explore for 10 min. The time for the experimental mouse spent in sniffing in the “interaction zone” (300 × 300 mm) was recorded by Video tracking software (Stoelting, USA).

**Forced swimming test**

Mice were placed in a cylinder of water (temperature 24–25 °C; 10 cm in diameter, 30 cm in height) for 10 min. The depth of water was set to prevent animals from touching the bottom with their hind limbs. Total immobility time was recorded by ANY-Maze Video Tracking System from the side, which was defined as floating or the least movement to maintain the head above the water.

**Supplementary Figures**

**
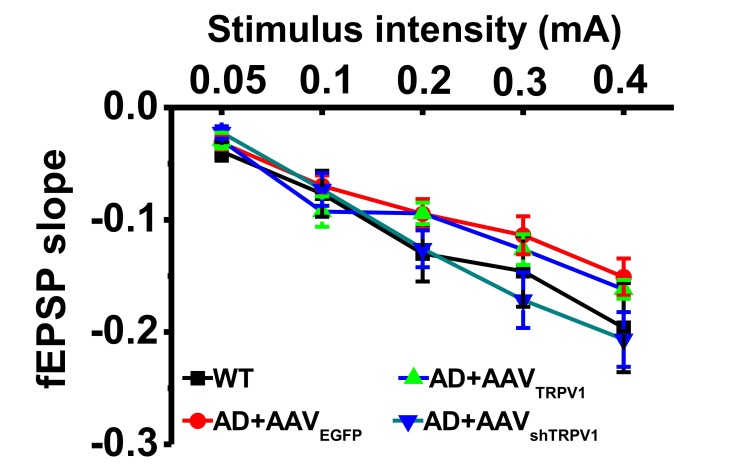
**

**Supplementary Fig. 1. TRPV1 does not alter the input-output relationship.** The fEPSP slope shows no significant difference at any stimulus intensity among these four groups (n = 5-6 in each group). Two-way ANOVA: F_(3,19)_ = 0.787, p = 0.517. One-way ANOVA: F_(3,19)_ = 1.078, p = 0.383 at 0.05 mA; F_(3,19)_ = 0.487, p = 0.696 at 0.1 mA; F_(3,19)_ = 1.378, p = 0.282 at 0.2 mA; F_(3,19)_ = 1.287, p = 0.309 at 0.3 mA; F_(3,19)_ = 1.231, p = 0.327 at 0.4 mA. Data are expressed as mean ± SEM.

**
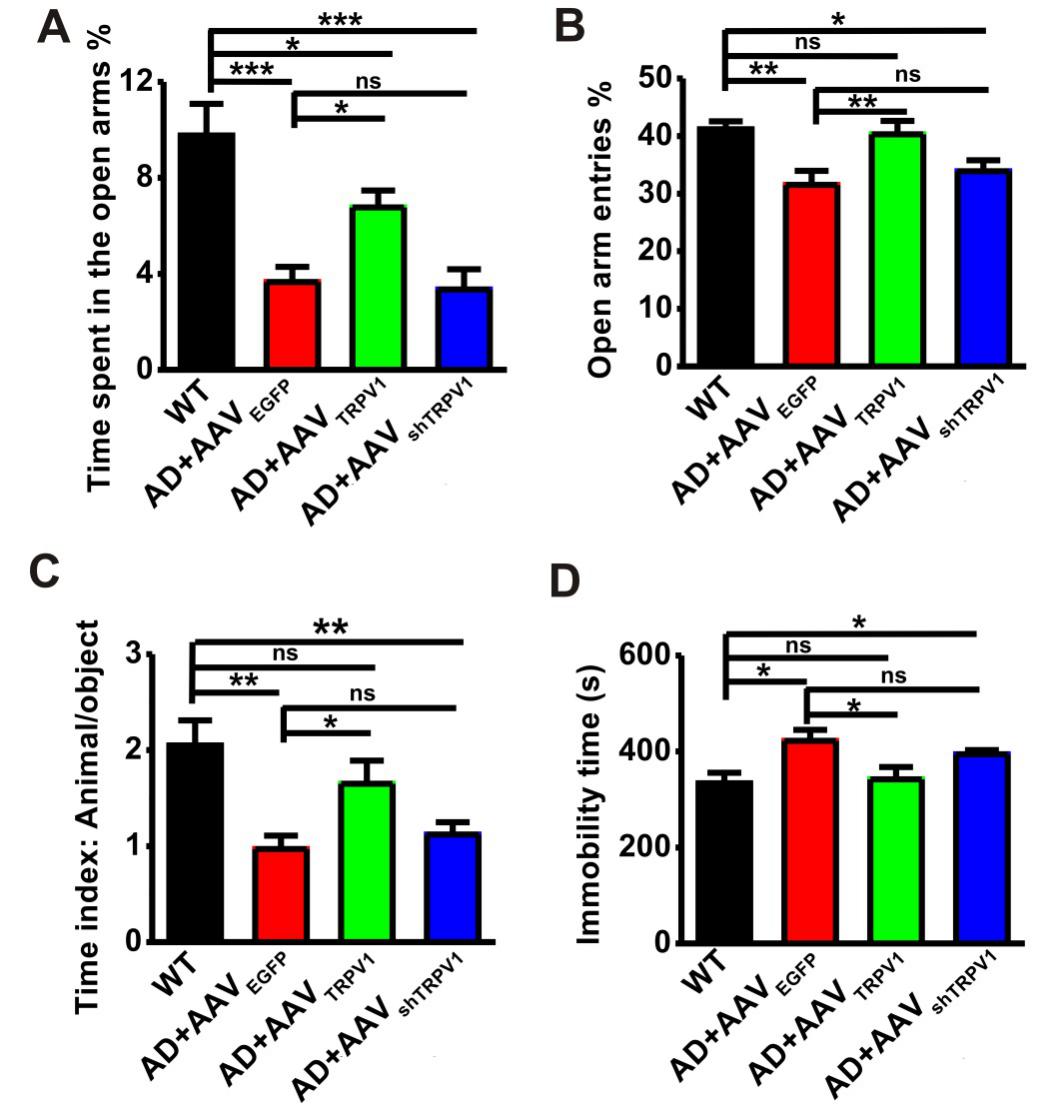
**

**Supplementary Fig. 2. TRPV1 ameliorates anxiety-/depressive-like behaviors in APP23/PS45 model mouse of AD.** The percentage of time spent in the open arms (A) and entries into open arms (B) during the elevated plus maze test (n = 7-8 in each group). One-way ANOVA: F_(3,26)_ = 10.380, p < 0.001 for time spent in the open arms; F_(3,26)_ = 5.490, p = 0.005 for number of entries into open arms. (C) The ratio of time to interact with the animal and object (n = 7-8 in each group). One-way ANOVA: F_(3,26)_ = 5.961, p = 0.003. (D) The immobility time during the force swimming test (n = 4-7 in each group). One-way ANOVA: F_(3,19)_ = 3.714, p = 0.029. Data are expressed as mean ± SEM, *p < 0.05, **p < 0.01, ***p < 0.001.
